# Diet-Dependent and Diet-Independent Hemorheological Alterations in Celiac Disease: A Case-Control Study

**DOI:** 10.14309/ctg.0000000000000256

**Published:** 2020-11-12

**Authors:** Zsolt Szakács, Beáta Csiszár, Mátyás Nagy, Nelli Farkas, Péter Kenyeres, Adrienn Erős, Alizadeh Hussain, Katalin Márta, Andrea Szentesi, Margit Tőkés-Füzesi, Tímea Berki, Áron Vincze, Kálmán Tóth, Péter Hegyi, Judit Bajor

**Affiliations:** 1 Institute for Translational Medicine, Medical School, University of Pécs, Pécs, Hungary;; 2 János Szentágothai Research Center, University of Pécs, Pécs, Hungary;; 3 Division of Cardiology, First Department of Medicine, Medical School, University of Pécs, Pécs, Hungary;; 4 Institute of Bioanalysis, Medical School, University of Pécs, Pécs, Hungary;; 5 Heim Pál National Insititute of Pediatrics, Budapest, Hungary;; 6 Division of Hematology, First Department of Medicine, Medical School, University of Pécs, Pécs, Hungary;; 7 Department of Laboratory Medicine, Medical School, University of Pécs, Pécs, Hungary;; 8 Department of Immunology and Biotechnology, Medical School, University of Pécs, Pécs, Hungary;; 9 Division of Gastroenterology, First Department of Medicine, Medical School, University of Pécs, Pécs, Hungary.

## Abstract

Hemorheology is the study of the flow properties of the blood and its elements, which, together with natural anticoagulants, are important determinants of cardiovascular events. This study aimed to assess hemorheological and natural anticoagulant profiles of patients with celiac disease (CeD) comprehensively.

**METHODS:**

Our study is a case-control study (registered under ISRCTN49677481) comparing patients with CeD with age- and sex-matched control subjects (1:1). We measured erythrocyte deformability (ED) at high (3–30 Pa) and low shears (0.3–3 Pa), erythrocyte aggregation, whole blood viscosity, plasma viscosity, and natural anticoagulants (protein C, protein S, and antithrombin activity). Adherence to gluten-free diet was estimated through dietary interview and urine gluten immunogenic peptide (urine GIP) detection.

**RESULTS:**

After matching, we analyzed the data of 100 study participants. ED at high shears was impaired in CeD (*P* < 0.05 for all shears, confirmed by random forest analysis) independently of findings on CeD-specific serological assessment and urine GIP detection but slightly dependently on dietary adherence (*P* = 0.025 for 30 Pa shear). ED at low shears seemed to be impaired only in urine GIP+ CeD patients (*P* < 0.05 for all comparisons with urine GIP− CeD patients and control subjects). All parameters describing erythrocyte aggregation and whole blood viscosity were shifted toward a prothrombotic direction in patients with CeD with poor dietary adherence compared with those with good dietary adherence. Plasma viscosity and activity of natural anticoagulants did not differ across groups.

**DISCUSSION:**

We observed diet-dependent and diet-independent prothrombotic hemorheological alterations in CeD, which can contribute to the elevated cardiovascular risk. The untoward metabolic changes during gluten-free diet, which can further aggravate hemorheological status, may indicate the implementation of prevention strategies.

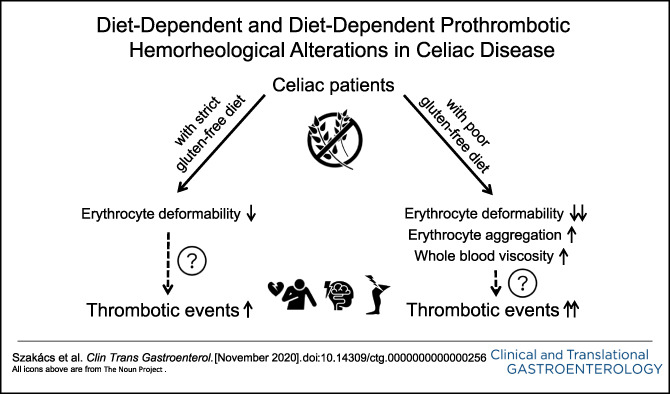

## INTRODUCTION

Celiac disease (CeD) is a chronic, immune-mediated disorder, which develops on gluten exposure in genetically susceptible individuals (1). CeD affects about 1% of the population; nevertheless, its prevalence is still increasing (2).

The disease carries the risk of severe complications including but not restricted to nutritional deficiencies, metabolic bone disease, and malignant tumors (1). Besides, cardiovascular (CV) comorbidities should be highlighted. A meta-analysis (2012) of population-based studies suggested an increased 1.19-fold risk of CV-related deaths for patients with CeD (3), confirmed by a recent (2020) population-based Swedish cohort study (4). The increased CV risk can be explained with several pathophysiological mechanisms. Prothrombotic alterations include malabsorption (vitamin K deficiency with subsequent protein C and protein S deficiency, or vitamin B_12_/B_9_ deficiency with subsequent hyperhomocysteinemia), thrombophilic antibodies, accelerated atherosclerosis, endothelial and platelet dysfunction, comorbid conditions (e.g., antiphospholipid syndrome), subclinical chronic inflammation, and genetics (5,6). Energy density and nutrient composition of gluten-free diet (GFD) often deviate from the optimal (7–10), raising concerns about a variety of modifiable CV risk factors to change unfavorably (11).

Besides the factors mentioned above, the hemorheological profile should be taken into account when potential prothrombotic alterations are investigated. Hemorheology (in other words, blood rheology) is the study of the flow properties of the blood and its elements. Hemorheological indicators, such as hematocrit (HTC), whole blood viscosity (WBV), plasma viscosity (PV), erythrocyte deformability (ED), and erythrocyte aggregation (EA), play fundamental roles in the maintenance of microcirculation (12–14). Besides, partly through increased endothelial shear stress (15), an altered hemorheological profile has long been known to be an essential determinant of thrombogenesis (16,17) and was reported in various immune-mediated diseases including systemic lupus erythematosus (18,19) and rheumatoid arthritis (19,20). Hemorheological indicators are extensively studied in inflammatory bowel disease; reports indicated (sometimes, activity dependent) alterations of ED, EA, fibrinogen, and PV in both Crohn's disease and ulcerative colitis (21–27). Nevertheless, little (or rather no) attention has been paid to hemorheology in CeD. Similarly, natural anticoagulants (protein C, protein S, and antithrombin), the physiological inhibitors of the coagulation cascade, and thrombogenesis were never investigated in patients with CeD in a comparative study.

Our aim was the comprehensive evaluation of hemorheological and natural anticoagulant profiles of patients with CeD with particular focus on the effects of dietary adherence to explore alterations which can contribute to the elevated CV risk.

## METHODS

The study was approved by the Regional and Local Research Ethics Committee (University of Pécs, Pécs, Hungary; Ref No. 6917) and registered in the ISRCTN Registry under registration number ISRCTN49677481. Full technical details of the protocol are published elsewhere (28). This report follows the STROBE Statement (29).

### Design, setting, and eligibility

Our study is a single-center observational study with a case-control design. Adults (aged ≥18 years) were recruited consecutively from the gastroenterology outpatient clinic of our academic hospital between June 2017 and May 2018. Cases were patients with biopsy-confirmed CeD diagnosed according to the current guidelines (30,31). Control subjects were individuals in whom CeD was excluded by the treating gastroenterologist specialist (30,31). Exclusion criteria from the study are detailed in the prestudy protocol (28).

### Outcomes

Outcomes included hemorheological parameters (describing HTC, WBV, PV, fibrinogen, ED, and EA) and the activity of natural anticoagulants (antithrombin, protein C, and protein S).

### Flow and timing

Potential participants with a definitive diagnosis were screened for eligibility by a gastroenterologist specialist. If consented to participate, detailed medical history was obtained, and medical files were revised; then, the questionnaires were administered by the same person. The visit ended with a dietary interview, followed by blood and urine collection in our central laboratory unit.

### Questionnaires, laboratory measurements, and dietary interview

We used a thrombophilia questionnaire to assess arterial and venous thrombotic risk factors (for the items of the questionnaire, see Supplementary Digital Content 1, http://links.lww.com/CTG/A418) and the Gastrointestinal Symptom Rating Scale to assess the severity of gastrointestinal symptoms (32).

Laboratory tests were performed from venous blood as detailed in the prestudy protocol (28). Hemorheology-related terms and measurements are detailed in Table 1. We adhered to the guidelines proposed by the International Expert Panel for Standardization of Hemorheological Methods during the tests (33).

**Table 1.**
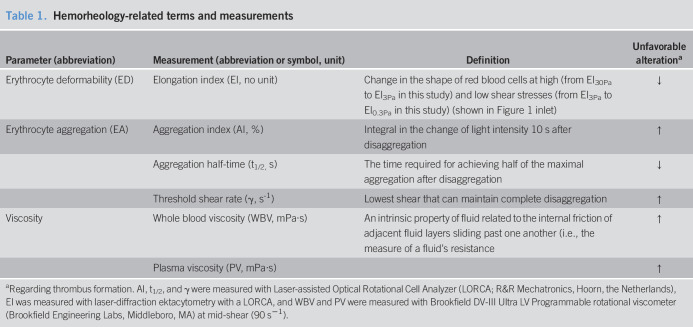
Hemorheology-related terms and measurements

Parameter (abbreviation)	Measurement (abbreviation or symbol, unit)	Definition	Unfavorable alteration^a^
Erythrocyte deformability (ED)	Elongation index (EI, no unit)	Change in the shape of red blood cells at high (from EI_30Pa_ to EI_3Pa_ in this study) and low shear stresses (from EI_3Pa_ to EI_0.3Pa_ in this study) (shown in Figure 1 inlet)	↓
Erythrocyte aggregation (EA)	Aggregation index (AI, %)	Integral in the change of light intensity 10 s after disaggregation	↑
	Aggregation half-time (t_1/2,_ s)	The time required for achieving half of the maximal aggregation after disaggregation	↓
	Threshold shear rate (γ, s^-1^)	Lowest shear that can maintain complete disaggregation	↑
Viscosity	Whole blood viscosity (WBV, mPa·s)	An intrinsic property of fluid related to the internal friction of adjacent fluid layers sliding past one another (i.e., the measure of a fluid's resistance	↑
	Plasma viscosity (PV, mPa·s)		↑

a Regarding thrombus formation. AI, t_1/2_, and γ were measured with Laser-assisted Optical Rotational Cell Analyzer (LORCA; R&R Mechatronics, Hoorn, the Netherlands), EI was measured with laser-diffraction ektacytometry with a LORCA, and WBV and PV were measured with Brookfield DV-III Ultra LV Programmable rotational viscometer (Brookfield Engineering Labs, Middleboro, MA) at mid-shear (90 s^−1^).

Urine gluten immunogenic peptides (urine GIPs) were measured by a point-of-care test according to the test's user guide (iVYCHECK GIP Urine, Biomedal, Spain).

In patients with CeD, dietary adherence was estimated through a dietary interview conducted by an accredited dietitian who judged adherence on a visual analog scale between 1 (regular gluten-containing diet) and 10 (perfect GFD). The same person ensured that control subjects did not follow a GFD.

### Subgroup analysis

Patients with CeD were divided by CeD-specific serology (based on tissue transglutaminase antibody [tTG]-IgA/IgG and endomysial antibody [EMA] IgA/IgG levels with the cutoff from the test's user guide), by estimated dietary adherence based on dietary interview (scores <8 points were chosen to indicate poor dietary adherence), and by the results of urine GIP measurement (interpreted according to the test's user guide). CeD-specific serology rather reflects the intensity of the ongoing immune response and is sensitive to detect major dietary transgressions, whereas urine GIP positivity rather reflects gluten intake of the past 2–3 days (34).

### Sample size and data analysis

Because hemorheological indicators were never determined in CeD, we planned to recruit 100 age- and sex-matched participants (1:1 ratio) in the first phase to determine further target numbers for the second phase (28). Completing the first phase, we realized that to reach the level of significance for the observed mean differences between patients with CeD and control subjects at α = 0.05 and β = 0.80, we should recruit an unfeasible number of subjects (>1,000 patients for EA indicators, 247 patients for PV, and 289 patients for WBV per group), so that we decided to stop the study for futility.

After matching by age (±5 years tolerance) and sex (±10% tolerance), descriptive statistics were performed. Categorical variables were given in proportions (% of total). Continuous variables were given with central tendencies (mean and/or median) and measure of dispersion (SD, quartiles, and/or range) based on distribution determined by the visual inspection of Q-Q plots. Logarithmic transformation was applied to normalize the distribution in the case of t_1/2_, γ, WBV, PV, antithrombin, and protein S activity.

In univariate analysis, we used the Welch, Mann-Whitney, χ^2^, and Fisher tests; one-way ANOVA (with the Tukey *post hoc* test), Kruskal-Wallis test (with the Mann-Whitney *post hoc* test) and Bonferonni correction (where appropriate)). In multivariate analysis, we used the random forest method to determine and graphically display the relative importance of each predictor. During prediction, 100 random forests with 500 trees in each were grown using conditional inference method to avoid bias toward dependent predictors and overfitting (35).

The calculations were performed with IBM SPSS for Windows (version 25.0 statistical software package; Armonk, NY: IBM Corp.) and R statistical language (version 3.6, party statistical software package; R Core Team, Vienna, Austria).

## RESULTS

### Characteristics of the study population

A total of 162 consecutive potential participants were screened for eligibility, 126 of which were included in the study (for flowchart, see Supplementary Digital Content 2, http://links.lww.com/CTG/A419). After matching patients by age and sex (1:1, n = 100), groups were similar in baseline characteristics except for cholesterol profile, eosinophil cell count, and red blood cell distribution width (Tables 2 and 3). For comorbid conditions and medications of the study participants, see Supplementary Digital Content 3, http://links.lww.com/CTG/A420.

**Table 2.**
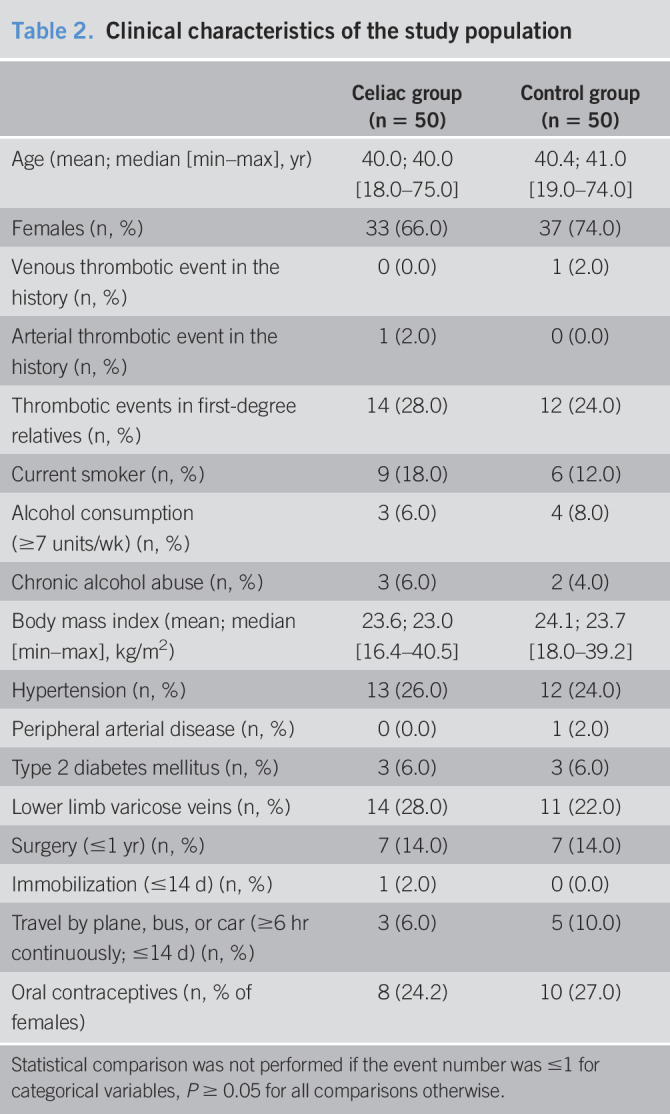
Clinical characteristics of the study population

	Celiac group (n = 50)	Control group (n = 50)
Age (mean; median [min–max], yr)	40.0; 40.0 [18.0–75.0]	40.4; 41.0 [19.0–74.0]
Females (n, %)	33 (66.0)	37 (74.0)
Venous thrombotic event in the history (n, %)	0 (0.0)	1 (2.0)
Arterial thrombotic event in the history (n, %)	1 (2.0)	0 (0.0)
Thrombotic events in first-degree relatives (n, %)	14 (28.0)	12 (24.0)
Current smoker (n, %)	9 (18.0)	6 (12.0)
Alcohol consumption (≥7 units/wk) (n, %)	3 (6.0)	4 (8.0)
Chronic alcohol abuse (n, %)	3 (6.0)	2 (4.0)
Body mass index (mean; median [min–max], kg/m^2^)	23.6; 23.0 [16.4–40.5]	24.1; 23.7 [18.0–39.2]
Hypertension (n, %)	13 (26.0)	12 (24.0)
Peripheral arterial disease (n, %)	0 (0.0)	1 (2.0)
Type 2 diabetes mellitus (n, %)	3 (6.0)	3 (6.0)
Lower limb varicose veins (n, %)	14 (28.0)	11 (22.0)
Surgery (≤1 yr) (n, %)	7 (14.0)	7 (14.0)
Immobilization (≤14 d) (n, %)	1 (2.0)	0 (0.0)
Travel by plane, bus, or car (≥6 hr continuously; ≤14 d) (n, %)	3 (6.0)	5 (10.0)
Oral contraceptives (n, % of females)	8 (24.2)	10 (27.0)

Statistical comparison was not performed if the event number was ≤1 for categorical variables, *P* ≥ 0.05 for all comparisons otherwise.

**Table 3.**
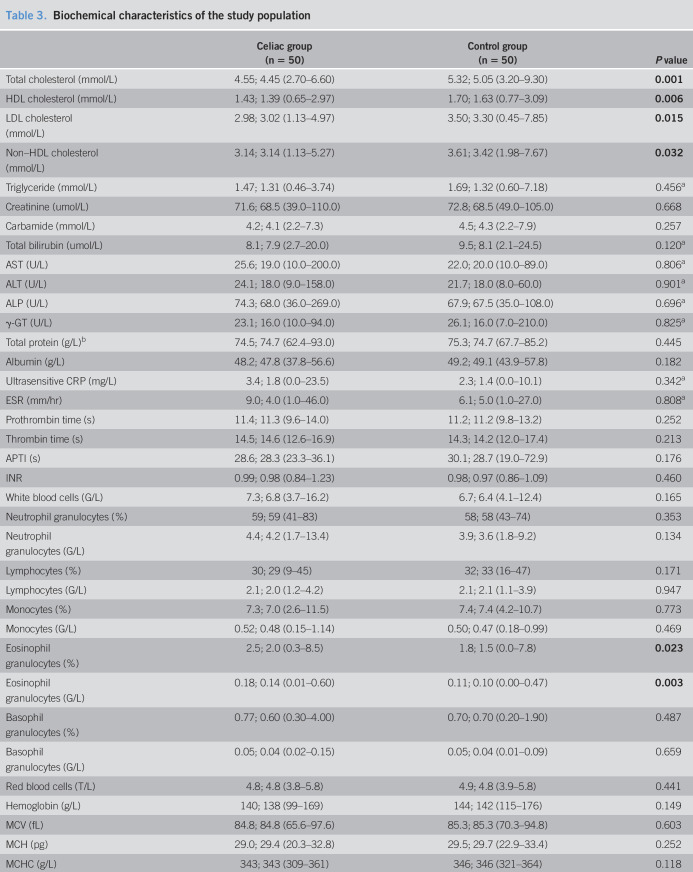

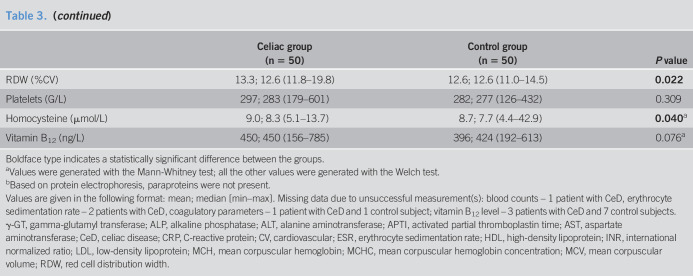
Biochemical characteristics of the study population

	Celiac group (n = 50)	Control group (n = 50)	*P* value
Total cholesterol (mmol/L)	4.55; 4.45 (2.70–6.60)	5.32; 5.05 (3.20–9.30)	**0.001**
HDL cholesterol (mmol/L)	1.43; 1.39 (0.65–2.97)	1.70; 1.63 (0.77–3.09)	**0.006**
LDL cholesterol (mmol/L)	2.98; 3.02 (1.13–4.97)	3.50; 3.30 (0.45–7.85)	**0.015**
Non–HDL cholesterol (mmol/L)	3.14; 3.14 (1.13–5.27)	3.61; 3.42 (1.98–7.67)	**0.032**
Triglyceride (mmol/L)	1.47; 1.31 (0.46–3.74)	1.69; 1.32 (0.60–7.18)	0.456^a^
Creatinine (umol/L)	71.6; 68.5 (39.0–110.0)	72.8; 68.5 (49.0–105.0)	0.668
Carbamide (mmol/L)	4.2; 4.1 (2.2–7.3)	4.5; 4.3 (2.2–7.9)	0.257
Total bilirubin (umol/L)	8.1; 7.9 (2.7–20.0)	9.5; 8.1 (2.1–24.5)	0.120^a^
AST (U/L)	25.6; 19.0 (10.0–200.0)	22.0; 20.0 (10.0–89.0)	0.806^a^
ALT (U/L)	24.1; 18.0 (9.0–158.0)	21.7; 18.0 (8.0–60.0)	0.901^a^
ALP (U/L)	74.3; 68.0 (36.0–269.0)	67.9; 67.5 (35.0–108.0)	0.696^a^
γ-GT (U/L)	23.1; 16.0 (10.0–94.0)	26.1; 16.0 (7.0–210.0)	0.825^a^
Total protein (g/L)^b^	74.5; 74.7 (62.4–93.0)	75.3; 74.7 (67.7–85.2)	0.445
Albumin (g/L)	48.2; 47.8 (37.8–56.6)	49.2; 49.1 (43.9–57.8)	0.182
Ultrasensitive CRP (mg/L)	3.4; 1.8 (0.0–23.5)	2.3; 1.4 (0.0–10.1)	0.342^a^
ESR (mm/hr)	9.0; 4.0 (1.0–46.0)	6.1; 5.0 (1.0–27.0)	0.808^a^
Prothrombin time (s)	11.4; 11.3 (9.6–14.0)	11.2; 11.2 (9.8–13.2)	0.252
Thrombin time (s)	14.5; 14.6 (12.6–16.9)	14.3; 14.2 (12.0–17.4)	0.213
APTI (s)	28.6; 28.3 (23.3–36.1)	30.1; 28.7 (19.0–72.9)	0.176
INR	0.99; 0.98 (0.84–1.23)	0.98; 0.97 (0.86–1.09)	0.460
White blood cells (G/L)	7.3; 6.8 (3.7–16.2)	6.7; 6.4 (4.1–12.4)	0.165
Neutrophil granulocytes (%)	59; 59 (41–83)	58; 58 (43–74)	0.353
Neutrophil granulocytes (G/L)	4.4; 4.2 (1.7–13.4)	3.9; 3.6 (1.8–9.2)	0.134
Lymphocytes (%)	30; 29 (9–45)	32; 33 (16–47)	0.171
Lymphocytes (G/L)	2.1; 2.0 (1.2–4.2)	2.1; 2.1 (1.1–3.9)	0.947
Monocytes (%)	7.3; 7.0 (2.6–11.5)	7.4; 7.4 (4.2–10.7)	0.773
Monocytes (G/L)	0.52; 0.48 (0.15–1.14)	0.50; 0.47 (0.18–0.99)	0.469
Eosinophil granulocytes (%)	2.5; 2.0 (0.3–8.5)	1.8; 1.5 (0.0–7.8)	**0.023**
Eosinophil granulocytes (G/L)	0.18; 0.14 (0.01–0.60)	0.11; 0.10 (0.00–0.47)	**0.003**
Basophil granulocytes (%)	0.77; 0.60 (0.30–4.00)	0.70; 0.70 (0.20–1.90)	0.487
Basophil granulocytes (G/L)	0.05; 0.04 (0.02–0.15)	0.05; 0.04 (0.01–0.09)	0.659
Red blood cells (T/L)	4.8; 4.8 (3.8–5.8)	4.9; 4.8 (3.9–5.8)	0.441
Hemoglobin (g/L)	140; 138 (99–169)	144; 142 (115–176)	0.149
MCV (fL)	84.8; 84.8 (65.6–97.6)	85.3; 85.3 (70.3–94.8)	0.603
MCH (pg)	29.0; 29.4 (20.3–32.8)	29.5; 29.7 (22.9–33.4)	0.252
MCHC (g/L)	343; 343 (309–361)	346; 346 (321–364)	0.118
RDW (%CV)	13.3; 12.6 (11.8–19.8)	12.6; 12.6 (11.0–14.5)	**0.022**
Platelets (G/L)	297; 283 (179–601)	282; 277 (126–432)	0.309
Homocysteine (µmol/L)	9.0; 8.3 (5.1–13.7)	8.7; 7.7 (4.4–42.9)	**0.040**^a^
Vitamin B_12_ (ng/L)	450; 450 (156–785)	396; 424 (192–613)	0.076^a^

Boldface type indicates a statistically significant difference between the groups.

a Values were generated with the Mann-Whitney test; all the other values were generated with the Welch test.

b Based on protein electrophoresis, paraproteins were not present.

Values are given in the following format: mean; median [min–max]. Missing data due to unsuccessful measurement(s): blood counts – 1 patient with CeD, erythrocyte sedimentation rate – 2 patients with CeD, coagulatory parameters – 1 patient with CeD and 1 control subject; vitamin B_12_ level – 3 patients with CeD and 7 control subjects.

γ-GT, gamma-glutamyl transferase; ALP, alkaline phosphatase; ALT, alanine aminotransferase; APTI, activated partial thromboplastin time; AST, aspartate aminotransferase; CeD, celiac disease; CRP, C-reactive protein; CV, cardiovascular; ESR, erythrocyte sedimentation rate; HDL, high-density lipoprotein; INR, international normalized ratio; LDL, low-density lipoprotein; MCH, mean corpuscular hemoglobin; MCHC, mean corpuscular hemoglobin concentration; MCV, mean corpuscular volume; RDW, red cell distribution width.

Patients with CeD were, on average, aged 31.9 years (range 0–73 years) at diagnosis. Three patients had not started GFD at the time of the study, and all the others were ≥1 year on GFD (median 5.5 years, range: 0.0–36.0 years). At the time of the study, 6 patients (12% of the total) tested positive for urine GIP, 10 patients (20% of the total) scored <8 points on the dietary interview (with median 9 points), and 14 patients (28% of the total) tested positive for tTG or EMA. Patients with CeD rated a median of 1.50 points on the Gastrointestinal Symptom Rating Scale.

Control subjects attended a regular checkup (n = 18), were admitted for investigation (n = 16) or mandatory occupational health assessment (n = 16). None of them followed a GFD, and all tested negative for both tTG and EMA.

### Hemorheological parameters in patients with celiac disease vs control subjects

**Figure 1.**
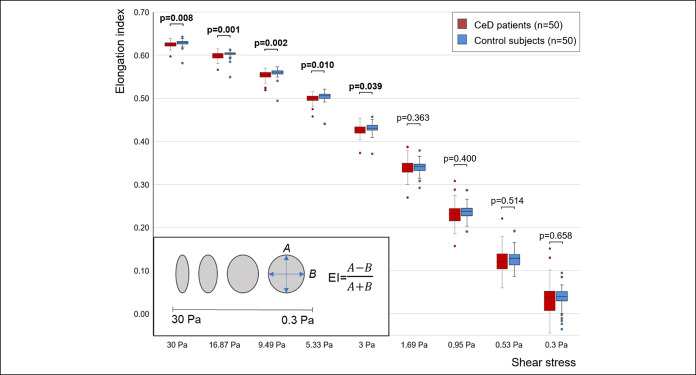
Erythrocyte deformability at different shears (ektacytogram) in patients with celiac disease and control subjects. The horizontal axis indicates the different shears from 0.3 to 30 Pa; the vertical axis shows the elongation index (EI, calculated based on the equation presented in the inlet). Measurements of EI were performed with LORCA. *P* values < 0.05 are highlighted with bold, and were generated with the Mann-Whitney test. Inlet: model for erythrocyte deformation at different shears describing the transition from biconcave to ellipsoid shape. In the equation, A and B represent the long and short axes of the cells, respectively, as indicated with blue arrows in the figure. EI, elongation index (no. of patients = 100).

**Figure 2.**
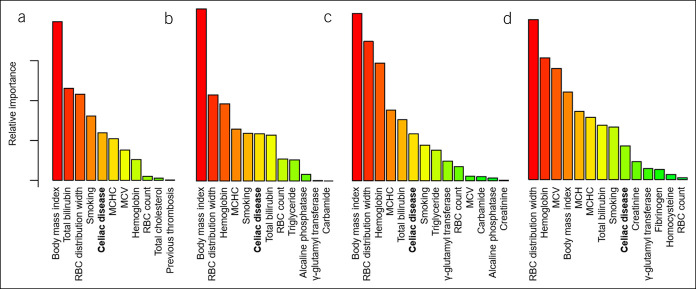
Important predictors of erythrocyte deformability represented by the elongation index at different shears. Panel A: 30 Pa; panel B: 16.87 Pa; panel C: 9.49 Pa; and panel D: 5.33 Pa. Celiac disease is highlighted with bold. The figure was generated with random forest analysis. We imputed 34 covariates, but only those above 0 are displayed because these should be considered important predictors of the outcome. The relative importance is proportional to the height of the bars. MCH, mean corpuscular hemoglobin; MCHC, mean corpuscular hemoglobin concentration; MCV, mean corpuscular volume; RBC, red blood cell (no. of patients = 97).

Patients with CeD had impaired ED at high shears compared with controls (*P* < 0.05 for the comparisons at 5 shears from 3 to 30 Pa; Figure 1), whereas we observed no significant difference in markers of EA, WBV, PV, and fibrinogen between the groups (for table, see Supplementary Digital Content 4, http://links.lww.com/CTG/A421, which describes the results of comparisons). After correction for 34 clinical and biochemical variables (including CeD vs control status), random forest analysis indicated that CeD is an important determinant of ED at high shears from 5.33 to 30 Pa (Figure 2), unlike at lower shears or regarding EA, HTC, WBV, and PV (for figure, see Supplementary Digital Content 5, http://links.lww.com/CTG/A422, which displays the results of random forest analysis). The most important predictors of hemorheological parameters overlapped those known from the literature, ensuring the validity of random forest analysis.

### Association of dietary adherence and serological findings with hemorheological parameters

Supplementary Digital Content 6 (http://links.lww.com/CTG/A423) summarizes raw data and statistics on findings on serological assessment, dietary interview, and urine GIP detection.

**Figure 3.**
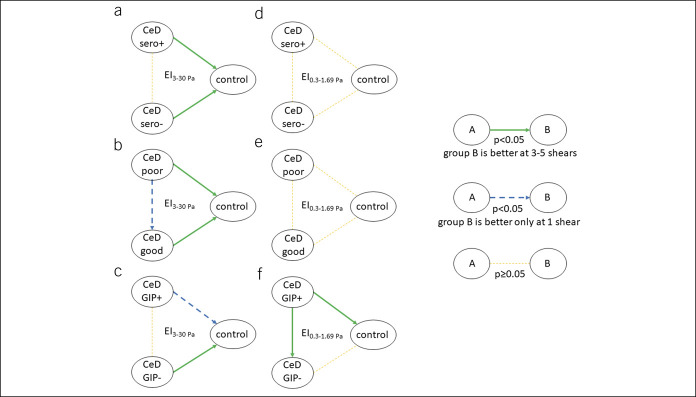
Association of erythrocyte deformability with the CeD-specific serology, adherence based on dietary interview, and urine GIP measurement. Erythrocyte deformability is represented by the elongation index. Panels A and D: serology (based on EMA-IgA/G and tTG-IgA/G) and erythrocyte deformability at low and high shears, respectively. Panels B and E: adherence estimated through dietary interview and erythrocyte deformability at low and high shears, respectively. Panels C and F: adherence estimated through urine GIP detection and erythrocyte deformability at low and high shears, respectively. *P* values were adjusted for multiplicity. Green solid lines represent *P* < 0.05 at 3–5 shears favoring the group at the arrow tip. Blue dashed lines represent *P* < 0.05 at 1 shear favoring the group at the arrow tip. Yellow dashed lines represent no significant difference between groups. CeD, celiac disease; EI, elongation index; EMA, endomysial antibody; GIP, gluten immunogenic peptide; tTG, tissue transglutaminase antibody (no. of patients = 100).

ED at high shears seemed to be impaired both in patients with CeD who were seropositive and seronegative (Figure 3a) and both in patients with CeD with poor and good dietary adherence (Figure 3B) compared with control subjects. However, ED at high shears did not differ significantly between seropositive and seronegative patients with CeD (Figure 3a), whereas those with good adherence had better ED only at EI_30pa_ compared with those with poor adherence (which was not supported by the results of urine GIP measurement) (Figure 3b,c). These suggest that the impairment in ED at high shears is independent of the EMA/tTG-mediated immune response and only partly dependent on dietary adherence (favoring a better GFD). Interestingly, ED at low shears did not differ across groups irrespective of findings on serological assessment and dietician-reported dietary adherence (Figure 3d,e). Opposingly, urine GIP+ CeD patients had significantly impaired ED compared with urine GIP− CeD patients and control subjects (Figure 3f) without a difference between urine GIP− CeD patients and control subjects. These results suggest that ED at low shears may not be influenced by EMA/tTG-mediated immune response but may be influenced by other effects of gluten or related proinflammatory reaction.

**Figure 4.**
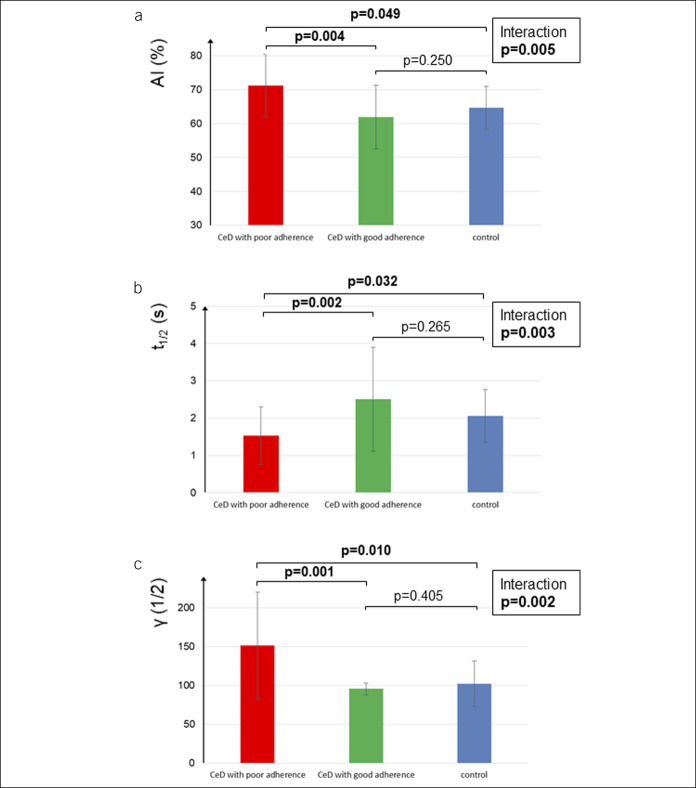
Association of erythrocyte aggregation with dietician-reported dietary adherence. Panel A: aggregation index across groups. Panel B: aggregation half-time across groups. Panel C: threshold shear rate across groups. *P* values were generated after logarithmic transformation of the data and were adjusted for multiplicity in the analysis. AI, aggregation index (no. of patients = 100); CeD, celiac disease.

EA seemed to be significantly impaired in patients with CeD with poor adherence compared with those with good adherence and control subjects (Figure 4). The association applies to AI, t_1/2_, and γ consistently (adjusted *P* values <0.01 for all; for figure, see Supplementary Digital Content 7, http://links.lww.com/CTG/A424), suggesting prothrombotic alterations in patients with CeD with poor adherence. However, seropositive patients did not differ from seronegative ones so that EMA/tTG-mediated immune response is unlikely explaining the results.

Although WBV seemed to be lower in patients with CeD with good adherence compared with patients with CeD with poor adherence, none of the groups differed significantly from control subjects. HTC, WBV, and PV did not seem to be different substantially across the groups. For figures, see Supplementary Digital Content 7, http://links.lww.com/CTG/A424.

### Natural anticoagulants

Measuring the endogenous anticoagulants, we observed no difference in protein C, protein S, and antithrombin activity between the CeD and control groups (Table 4), while CeD proved not to be an important predictor in random forest analysis. Dividing patients by findings on serological assessment, dietician-reported dietary adherence, or by results of urine GIP measurement did not reveal any difference across the groups.

**Table 4.**
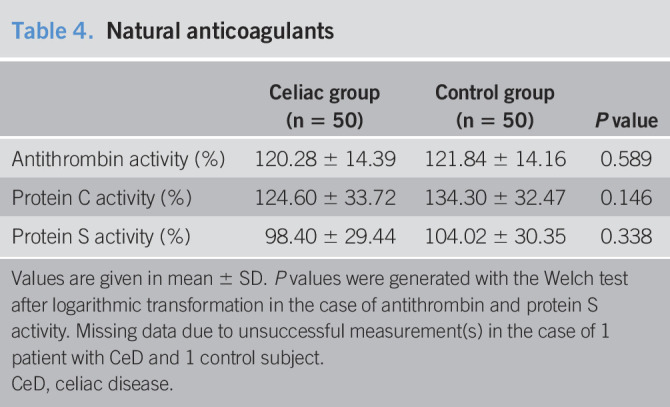
Natural anticoagulants

	Celiac group (n = 50)	Control group (n = 50)	*P* value
Antithrombin activity (%)	120.28 ± 14.39	121.84 ± 14.16	0.589
Protein C activity (%)	124.60 ± 33.72	134.30 ± 32.47	0.146
Protein S activity (%)	98.40 ± 29.44	104.02 ± 30.35	0.338

Values are given in mean ± SD. *P* values were generated with the Welch test after logarithmic transformation in the case of antithrombin and protein S activity. Missing data due to unsuccessful measurement(s) in the case of 1 patient with CeD and 1 control subject.

CeD, celiac disease.

## DISCUSSION

We identified impaired ED in CeD, which proved to be independent of findings on serological assessment and dependent, though only partly, on dietary adherence. Also, we found prothrombotic alterations in markers describing EA (AI, t_1/2_, and γ), especially in patients with poor dietary adherence. In addition, we observed lower WBV in patients with CeD with good dietary adherence compared with those with poor adherence. These findings contribute to the understanding of the mechanism of increased CV risk in CeD despite the absence of traditional risk factors: those with CeD have a lower body mass index, less smoking, lower diastolic blood pressure, and a lower level of cholesterol (36,37).

ED at high shears (3–30 Pa) rather models the pressure in the small arteries and arterioles, whereas ED at low shears (0.3–3 Pa) rather models that in the microcirculation. Rigid red blood cells (RBCs) may increase total peripheral resistance and require a higher pressure gradient to squeeze through capillaries. ED is determined mainly by membrane fluidity and cytoplasmatic viscosity, but extracorpuscular factors, such as splenic function or mechanical stress in the narrow capillaries, may have an irreversible impact on RBCs' membrane (14,38). A potential explanation for the impaired ED in CeD might be functional hyposplenism (affecting 16–77% of patients with CeD) when rigid RBCs are no longer removed from the circulation (39,40). Another option may be increased intracellular oxidative stress and reduced nitric oxide production (41). ED might be influenced by comorbidities and lifestyle factors as well (38), however, matching balanced major covariates between the groups. Of note, patients with CeD had lower total and low-density lipoprotein cholesterol levels in our study and previous research (37). Membrane cholesterol content is known to correlate with ED inversely (i.e., the higher the cholesterol, the more rigid the RBCs are) (42). Surprisingly, patients with CeD had impaired ED despite the lower cholesterol level (which is the so-called opposing bias supporting a cause-effect relationship), implying that from a hemorheological aspect, a lower cholesterol level should be considered optimal in CeD. This highlights the need for optimization of calorie density and suboptimal lipid profile of GFD.

Reports showed that an impaired ED is often found with CV diseases (43–45). In our study, at high shears, ED was impaired compared with control irrespective of seropositivity or dietary adherence, whereas patients with good dietician-reported adherence had better ED only at 30 Pa compared with those with poor dietary adherence (based on the dietary interview). This suggests that the impairment is independent on EMA/tTG-mediated immune reactions and only loosely dependent on gluten intake. Interestingly, GIP+ CeD patients, compared with GIP− CeD patients, had impaired ED at low shears, whereas we did not observe any difference across the groups if the patients were divided by findings on serological assessment or by the results of dietary interview. Theoretically, gluten, alone or via the activation of an immune response, may cause endothelial damage/activation via oxidative stress or other mechanisms (41,46,47), which can contribute to the mechanical damage of RBCs when passing through capillaries. In addition, altered endothelium-RBC-platelet interactions can facilitate thrombus formation. The associations about the impaired ED were established in a relatively young population. Considering age- and comorbidity-related changes (e.g., the metabolic syndrome, often seen in patients with CeD on GFD nowadays), further impairment of ED is expected with aging, contributing to the increased CV risk. This warns us of the importance of prevention and treatment of conditions associated with higher CV risk, such as the metabolic syndrome, in CeD. Nevertheless, low-grade (often conflicting) evidence from human clinical trials suggests that physical exercise (48), cholesterol-lowering drugs (42,49), fish oil (50), or polyphenols (51,52) can favorably change ED. Besides, encouragement of patients with CeD to commit to a healthy lifestyle (e.g., quitting smoking and maintaining optimal body weight) should become an organic part of counseling at regular follow-up visits.

In line with ED, EA is essential to maintain normal microcirculation and thrombus formation. EA is mainly determined by plasmatic (fibrinogen and other proteins) and cellular factors (e.g., membrane proteins) (12). Patients with CeD with poor dietary adherence showed prothrombotic alteration, but it seems that patients with CeD can restore normal EA with a strict GFD. Because GIPs, the derivatives of gliadin, are the triggers of the prothrombotic immune response in CeD, dietary transgressions might lead to a release in inflammatory proteins, known to affect EA (53). Because seropositivity does not seem to affect EA, this reaction may be independent of the EMA/tTG-mediated immune response. The prothrombotic changes in EA may be manifested in an increased WBV in patients with CeD with poor dietary adherence compared with those with good dietary adherence (based on the dietary interview). At the same time, HTC and fibrinogen, as important determinants of WBV, were similar across groups. Importantly, WBV was implicated to be associated with CV-related mortality (13).

The role of natural anticoagulants in thrombus formation is beyond dispute (54). Although we observed a lower activity of protein C and protein S compared with control subjects, the difference did not attain statistical significance, nor in subgroup analyses by serological assessment or dietician-reported dietary adherence. Interestingly, lupus anticoagulant was confirmed positive in 6 patients with CeD and 7 control subjects, and only sporadic cases were positive for antibodies against β2-glycoprotein, prothrombin, and cardiolipin, not supporting the theory about thrombophilic autoantibodies in CeD (6).

Strengths of the study include its novelty: to our best knowledge, no comparative study has assessed the hemorheological and natural anticoagulant profile of patients with CeD. In addition, the rigorous, standard methodology and comprehensive analysis should be mentioned. Our multimodal approach of dietary adherence included a dietary interview, celiac-specific serology, and measurement of urine GIP. Nevertheless, we must mention several limitations of the evidence. Prospective cohort studies recording the changes from diagnosis until follow-up visit provide better evidence of a cause-effect relationship. Per the recent guidelines, we do not perform follow-up biopsy routinely, and, consequently, we were unable to test the associations between intestinal histology and test results. However, mucosal changes do not necessary reflect dietary adherence (1,55) and are not necessarily associated with long-term CV outcomes (56).

Our conclusions are rather generalizable to younger and treated patients with CeD since 47 of 50 cases were on GFD ≥1 year (as reflected by the restored homocysteine level). It must be noted, however, that the length of GFD seems not important regarding our outcomes based on the results of random forest analyses.

We found impaired ED in CeD, which seemed to be independent of findings on serological assessment and only partially dependent on dietary adherence. Patients with CeD with poor dietary adherence exhibited prothrombotic alterations of EA, whereas HTC, WBV, PV, and natural anticoagulants seemed not to be substantially affected in CeD irrespective of dietary adherence. These findings should be validated in prospective cohort studies.

The unfavorable alterations of ED and EA can contribute to the elevated CV risk and highlight the importance of CV prevention in CeD during GFD in which good dietary adherence is of utmost importance. As metabolic syndrome has become a threat in CeD and its components (obesity, hypertension, diabetes mellitus, and dyslipidemia) can further aggravate hemorheological status, lifestyle changes (e.g., regular exercise and energy-optimized diet) or even pharmacological interventions (e.g., polyphenols and statins) may help to mitigate hemorheological alterations and thereby reduce CV risk. Randomized studies are called for to validate the clinical implications of our findings.

## CONFLICTS OF INTEREST

**Guarantor of the article:** Judit Bajor, MD, PhD.

**Specific author contributions:** Z.S., P.H., and J.B. conceptualized the study and drafted the manuscript. J.B. and Á.V. formally screened and consented study participants. Z.S., A.E., B.C., M.N., and K.M. administered the questionnaires and collected and validated the data. Measurements were performed and interpreted by B.C., P.K., and K.T. regarding hemorheological parameters, by A.H. and M.T.F. regarding hemostatic parameters, and by T.B. regarding immunological indicators. N.F. performed the statistical analysis. A.S. and K.M. coordinated the project. Á.V., K.T., P.H., and J.B. supervised the project. All authors revised the draft of the manuscript.

**Financial support:** This study was supported by an Economic Development and Innovation Operative Programme Grant (GINOP 2.3.2.-15-2016-00048) and a Human Resources Development Operative Programme Grant (EFOP-3.6.2-16-2017-00006) of the National Research, Development and Innovation Office, Hungary, and by the ÚNKP-18-3-I and ÚNKP-19-3-I New National Excellence Program of the Ministry of Human Capacities, Hungary.

**Potential competing interests:** None to report.

**Trial registration number:** ISRCTN49677481, ISRCTN Registry.

**Study Highlights**

**WHAT IS KNOWN**

✓ Risk of thrombotic events is higher among patients with celiac disease.
✓ Hemorheological and hemostatic alterations are potential contributors to cardiovascular events.

**WHAT IS NEW HERE**

✓ Erythrocyte deformation is impaired in patients with celiac disease, which is only partly dependent on dietary adherence.
✓ Erythrocyte aggregation is shifted toward a prothrombotic direction in patients with celiac disease with poor dietary adherence.

**TRANSLATIONAL IMPACT**

✓ The prothrombotic hemorheological alterations highlight the importance of cardiovascular prevention in celiac disease.
✓ Cardiovascular risk factors (e.g., metabolic syndrome) developing during gluten-free diet may further aggravate hemorheological status.
✓ Good dietary adherence can improve hemorheological alterations, but some parameters are independent of diet.

## Supplementary Material

SUPPLEMENTARY MATERIAL
