# Supplementary material for: Diet-Dependent and Diet-Independent Hemorheological Alterations in Celiac Disease: A Case-Control Study
Source: Clin Transl Gastroenterol. 2020 Nov 12;11(11):e00256. doi: 10.14309/ctg.0000000000000256 (PMC7665261; doi:10.14309/ctg.0000000000000256)
Supplement: SUPPLEMENTARY MATERIAL [file ct9-11-e00256-s001.docx]

**Supplemental Digital Content 1. Items of thrombophilia questionnaire**

Items were chosen based on the review article of Samuel et al. [1] and Previtali et al. [2].

• sex, age

• history of arterial/venous thrombotic events, thromboembolism, hereditary thrombophilias

• family history of arterial/venous thrombotic events, thromboembolism, hereditary thrombophilias (first-degree relatives)

• current medications (with particular emphasis on anticoagulants/antiplatelet agents)

• gynaecological history (with particular emphasis on pregnancy, pregnancy complications and abortions, hormone replacement therapy, oral contraceptives)

• smoking, alcohol and drug abuse

• comorbidities [with particular emphasis on malignant tumours, hypertension, diabetes mellitus, obesity (body mass index was calculated), cardiovascular, respiratory and kidney diseases, peripheral occlusive arterial disease, lipid metabolism disorders, immune-mediated disorders) and surgical history

• immobilization (bedrest >3 days), trauma, plaster cast in the past three months

• long travels (>6 hours) by car, plane or bus in a continuous sitting position

• lower limb varicose veins or chronic venous insufficiency

• acute infection in the past two weeks (with particular emphasis on common respiratory, urinary or gastrointestinal symptoms)

• invasive diagnostic or therapeutic intervention in the past two weeks

The questionnaire was completed during a guided interview by a medical doctor (who revised the patient’s printed and electronic medical files as well).

1. Goldhaber SZ. Risk factors for venous thromboembolism. Journal of the American College of Cardiology. 2010; 56(1):1-7. DOI: 10.1016/j.jacc.2010.01.057. PubMed PMID: 20620709.

2. Previtali E, Bucciarelli P, Passamonti SM, Martinelli I. Risk factors for venous and arterial thrombosis. Blood transfusion = Trasfusione del sangue. 2011; 9(2):120-38. Epub 10/25. doi: 10.2450/2010.0066-10. PubMed PMID: 21084000.
