# Supplementary figures and images for: Diet-Dependent and Diet-Independent Hemorheological Alterations in Celiac Disease: A Case-Control Study
Source: Clin Transl Gastroenterol. 2020 Nov 12;11(11):e00256. doi: 10.14309/ctg.0000000000000256 (PMC7665261; doi:10.14309/ctg.0000000000000256)

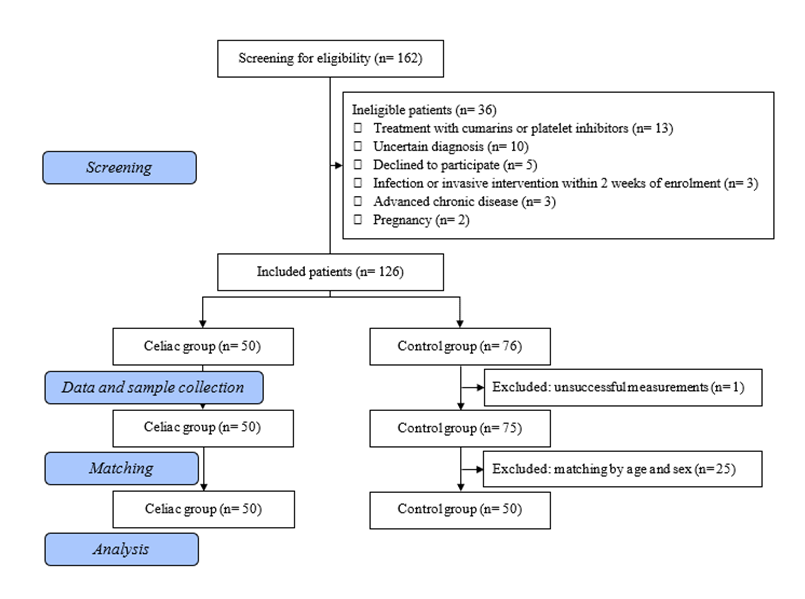

Supplement: SUPPLEMENTARY MATERIAL [file ct9-11-e00256-s002.tif]
