# Supplementary material for: Diet-Dependent and Diet-Independent Hemorheological Alterations in Celiac Disease: A Case-Control Study
Source: Clin Transl Gastroenterol. 2020 Nov 12;11(11):e00256. doi: 10.14309/ctg.0000000000000256 (PMC7665261; doi:10.14309/ctg.0000000000000256)
Supplement: SUPPLEMENTARY MATERIAL [file ct9-11-e00256-s003.docx]

**Supplemental Digital Content 2. Comorbid conditions and medications**

| **Subject identifier** | **Comorbid conditions** | **Medications** |
| --- | --- | --- |
| HER001 | Raynaud syndrome, sicca syndrome | Pantoprazole |
| HER002 | Hypertension, hypothyreosis, hyperprolactinaemia, iron deficiency, metrorrhagia, infertility, Turner syndrome (mosaic), retrocelebellar cyst | Iron replacement therapy, levothyroxine, carvedilol, bromocryptine, hesperidin-diosmin |
| HER003 | Hypertension, dermatitis herpetiformis | Dapsone |
| HER004 | Bronchial asthma, allergy, osteopenia | Desloratadin, budesonide-formoterol inhaler |
| HER005 | Hypertension | None |
| HER006 | Dermatitis herpetiformis, infertility | None |
| HER008 | Osteopenia, infertility | None |
| HER012 | Hypertension, sarcoidosis, glaucoma, seborrhea capitis, dermatitis herpetiformis | Telmisartan, norethisteron |
| HER013 | Hypertension, hypothyreosis, osteoporosis, dermatitis herpetiformis | Perindopril, levothyroxine, zolpidem |
| HER014 | Bronchial asthma, benign prostate hyperplasia, type 2 diabetes mellitus | Ciclesonide, tamsulosin, dutasteride |
| HER015 | Osteopenia | None |
| HER016 | Primary biliary cholangitis, osteoporosis | Ursodeoxycholic acid, pantoprazol |
| HER017 | None | None |
| HER018 | Strabism, hereditary hearing loss | None |
| HER019 | Hypertension, anxiety, osteopenia, hypothyreosis, allergic rhinitis | Moxonidine, amlodipine, duloxetine, levothyroxine |
| HER020 | Thoracic outlet syndrome, endometriosis, preeclampsia | Iron replacement therapy |
| HER021 | Oligoarthritis (atypical), alopecia areata, osteoporosis, corrected ventricular septal defect | None |
| HER022 | Osteopenia, dysmenorrhea | None |
| HER024 | None | None |
| HER025 | Hypertension, NAFLD | Valsartan |
| HER026 | None | None |
| HER029 | Anaemia, hyperthyroidism | None |
| HER031 | Airway allergy, dysmenorrhea | None |
| HER032 | None | None |
| HER033 | GERD, dermatitis herpetiformis | Omeprazole |
| HER034 | GERD | Esomeprazole |
| HER037 | None | None |
| HER038 | Hypertension, GERD, osteoporosis | Lisinopril, famotidine |
| HER039 | Hypertension, osteoporosis | Telmisartan, nebivolol |
| HER040 | None | None |
| HER041 | Benign tumour of the spleen and the small bowel, osteoporosis | Betahistine, alprazolam, vinpocetine, nicergoline, pantoprazole |
| HER042 | Metal allergy | None |
| HER043 | Food and pollen allergy | None |
| HER044 | Sjögren’s syndrome, autoimmune hepatitis and primary biliary cholangitis overlap | None |
| HER045 | Hypertension, osteopenia | Perindopril, naproxen, allopurinol |
| HER046 | Pollen allergy | None |
| HER047 | Hypertension, dermatitis herpetiformis, acute myocardial infarction (old) | Clonazepam, carvedilol, perindopril, indapamid, escitalopram, clopidogrol, mirtazapin, montelucast, formoterol-beclometasone inhaler |
| HER048 | None | None |
| HER050 | None | Nebivolol |
| HER051 | Bronchial asthma, lactose intolerance | Budesonide-formoterol inhaler |
| HER053 | Hypertension, small-fibre neuropathy | adjuvant analgetics (unknown) |
| HER054 | None | None |
| HER055 | None | None |
| HER057 | Psoriasis, lichen simplex, type 2 diabetes mellitus | Insulin, iron replacement therapy |
| HER058 | None | None |
| HER059 | None | None |
| HER060 | None | None |
| HER061 | Thyroid adenoma (post-stumectomy), cataract, benign prostate hyperplasia, type 2 diabetes mellitus | Alfulosizin, antidiabetic therapy (unknown) |
| HER062 | Hypertension | Perindopril, amlodipin |
| HER063 | None | Antihistamine (unknown) |
| HER201 | GERD, infertility, cervix carcinoma (operated on it >five years age, no relapse) | Rabeprazole |
| HER202 | Hypertension, GERD | Valsartan |
| HER203 | GERD | None |
| HER205 | Hypertension | Nebivolol |
| HER206 | Anxiety, GERD, small intestinal bacterial overgrowth (cured) | Bisoprolol, duloxetine, alprazolam |
| HER207 | None | None |
| HER208 | Graves’s disease | Methotyrine |
| HER209 | Pollen allergy | Desloratadine |
| HER210 | None | None |
| HER211 | None | None |
| HER212 | None | None |
| HER213 | None | None |
| HER214 | Hypertension, bronchial asthma, chronic kidney disease (mild with nearly normal creatinine), age-related macula degeneration, benign prostate hyperplasia | Allopurinol, atorvastatin, budesonide-formoterol inhaler, pantoprazole, perindopril |
| HER215 | Hypertension | Bisoprolol, clopamide |
| HER218 | Migraine | None |
| HER219 | GERD | Lansoprazole |
| HER220 | None | Bisoprolol |
| HER221 | Psoriasis | None |
| HER222 | Hypertension, glaucoma, diverticulosis | Valsartan |
| HER223 | Chronic pancreatitis, hypertension, Hashimoto-thyroiditis, bronchial asthma, rheumatoid arthritis, pulmonary embolism (old) | Valsartan, hydrochlorothiazide, levothyroxine, allopurinol, budesonide-formoterol inhaler |
| HER224 | Hashimoto-thyroiditis, occlusive peripheral artery disease, hypertension, type 2 diabetes mellitus | Famotidine, duloxetine, levothyroxine, irbesartan, bisoprolol, simvastatin, folic acid, alprazolam, insulin analogue antidiabetic drug (unknown) |
| HER225 | Hypertension, preeclampsia | Nebivolol, perindopril |
| HER226 | Adenomatous colon polyp, hypertension, type 2 diabetes mellitus | Bisoprolol, rosuvastatin, tioctanic acid, pantoprazol, telmisartan |
| HER228 | None | None |
| HER230 | Lactose intolerance | None |
| HER232 | Wilson’s disease | Penicillamine |
| HER233 | Bronchial asthma, hypertension, type 2 diabetes mellitus | Metformin, montelucast, ciclesonid inhaler, ramipril, amlodipin, nebivolol |
| HER234 | None | None |
| HER239 | Cryptogenic cirrhosis (Child A) | None |
| HER240 | None | None |
| HER241 | None | None |
| HER242 | Hypertension | None |
| HER244 | NAFLD | None |
| HER245 | None | None |
| HER246 | Airway allergy | None |
| HER247 | None | None |
| HER248 | Hypertension | Losartan |
| HER249 | None | None |
| HER250 | None | None |
| HER253 | Hashimoto-thyreoiditis | Levothyroxine |
| HER254 | Allergic rhinitis | Desloratadine |
| HER255 | Psoriasis, lactose intolerance | None |
| HER257 | None | None |
| HER258 | None | None |
| HER260 | Depression, migraine, goitre (non-functioning), GERD | Alprazolam, sertraline, desloratadine, iron replacement therapy |
| HER262 | Acne vulgaris | None |
| HER264 | Lactose intolerance, airway allergy | None |
| HER270 | None | None |
| HER272 | None | None |
| HER273 | None | None |

The subject identifiers were given before eligibility testing and matching; therefore, they are not necessarily consecutive. GERD: gastroesophageal reflux disease; NAFLD: non-alcoholic fatty liver disease.
