# Supplementary material for: Diet-Dependent and Diet-Independent Hemorheological Alterations in Celiac Disease: A Case-Control Study
Source: Clin Transl Gastroenterol. 2020 Nov 12;11(11):e00256. doi: 10.14309/ctg.0000000000000256 (PMC7665261; doi:10.14309/ctg.0000000000000256)
Supplement: SUPPLEMENTARY MATERIAL [file ct9-11-e00256-s004.docx]

**Supplemental Digital Content 3. Erythrocyte aggregation and viscosity**

|  | **Celiac group (n=50)** | **Control group (n=50)** | **p-value** |
| --- | --- | --- | --- |
| Hematocrit (%) | 43.3 ± 3.6 | 44.4 ± 3.3 | 0.117 |
| Whole blood viscosity (mPa·s) | 4.04 ± 0.43 | 4.14 ± 0.43 | 0.347 |
| Plasma viscosity (mPa·s) | 1.24 ± 0.16 | 1.27 ± 0.15 | 0.209 |
| Fibrinogen (g/L) | 2.90 [2.59–3.70] | 3.16 [2.71–3.59] | 0.948^*^ |
| Erythrocyte aggregation | | | |
| M | 5.34 ± 1.41 | 5.82 ± 1.26 | 0.076 |
| M1 | 12.22 ± 2.59 | 12.98 ± 2.33 | 0.127 |
| AI (%) | 63.8 ± 10.0 | 64.6 ± 6.3 | 0.613 |
| T_1/2_ (sec) | 2.31 ± 1.35 | 2.06 ± 0.71 | 0.677 |
| γ (1/sec) | 106.9 ± 50.0 | 102.3 ± 29.6 | 0.951 |

Values are given in mean ± standard deviation. *Values were generated with Mann-Whitney test; all the other values were generated with Welch test.
