# Supplementary material for: Diet-Dependent and Diet-Independent Hemorheological Alterations in Celiac Disease: A Case-Control Study
Source: Clin Transl Gastroenterol. 2020 Nov 12;11(11):e00256. doi: 10.14309/ctg.0000000000000256 (PMC7665261; doi:10.14309/ctg.0000000000000256)
Supplement: SUPPLEMENTARY MATERIAL [file ct9-11-e00256-s005.pptx]

## Slide 1
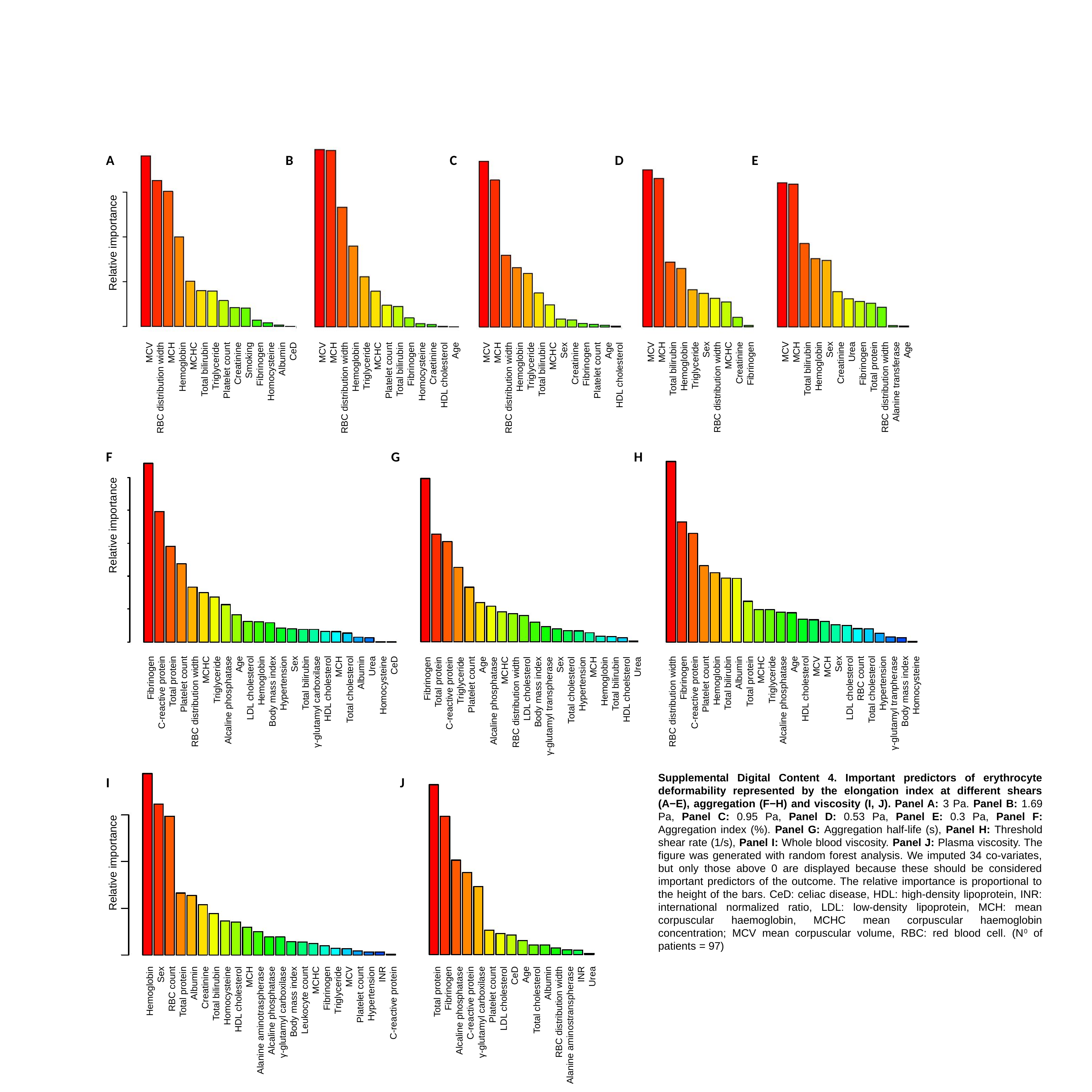

A
B
C
D
E
Relative importance
MCV
MCH
Total bilirubin
Hemoglobin
Triglyceride
Sex
RBC distribution width
MCHC
Creatinine
Fibrinogen
MCV
MCH
Total bilirubin
Hemoglobin
Sex
Creatinine
Urea
Fibrinogen
Total protein
RBC distribution width
Alanine transferase
Age
MCV
RBC distribution width
MCH
Hemoglobin
MCHC
Total bilirubin
Triglyceride
Platelet count
Creatinine
Smoking
Fibrinogen
Homocysteine
Albumin
CeD
MCV
MCH
RBC distribution width
Hemoglobin
Triglyceride
MCHC
Platelet count
Total bilirubin
Fibrinogen
Homocysteine
Craetinine
HDL cholesterol
Age
MCV
MCH
RBC distribution width
Hemoglobin
Triglyceride
Total bilirubin
MCHC
Sex
Creatinine
Fibrinogen
Platelet count
Age
HDL cholesterol
F
G
H
Relative importance
Fibrinogen
C-reactive protein
Total protein
Platelet count
RBC distribution width
MCHC
Triglyceride
Alcaline phosphatase
Age
LDL cholesterol
Hemoglobin
Body mass index
Hypertension
Sex
Total bilirubin
γ-glutamyl carboxilase
HDL cholesterol
MCH
Total cholesterol
Albumin
Urea
Homocysteine
CeD
RBC distribution width
Fibrinogen
C-reactive protein
Platelet count
Hemoglobin
Total bilirubin
Albumin
Total protein
MCHC
Triglyceride
Alcaline phosphatase
Age
HDL cholesterol
MCV
MCH
Sex
LDL cholesterol
RBC count
Total cholesterol
Hypertension
γ-glutamyl tranpherase
Body mass index
Homocysteine
Fibrinogen
Total protein
C-reactive protein
Triglyceride
Platelet count
Age
Alcaline phosphatase
MCHC
RBC distribution width
LDL cholesterol
Body mass index
γ-glutamyl transpherase
Sex
Total cholesterol
Hypertension
MCH
Hemoglobin
Total bilirubin
HDL choelsterol
Urea
Supplemental Digital Content 4. Important predictors of erythrocyte deformability represented by the elongation index at different shears (A−E), aggregation (F−H) and viscosity (I, J). Panel A: 3 Pa. Panel B: 1.69 Pa, Panel C: 0.95 Pa, Panel D: 0.53 Pa, Panel E: 0.3 Pa, Panel F: Aggregation index (%). Panel G: Aggregation half-life (s), Panel H: Threshold shear rate (1/s), Panel I: Whole blood viscosity. Panel J: Plasma viscosity. The figure was generated with random forest analysis. We imputed 34 co-variates, but only those above 0 are displayed because these should be considered important predictors of the outcome. The relative importance is proportional to the height of the bars. CeD: celiac disease, HDL: high-density lipoprotein, INR: international normalized ratio, LDL: low-density lipoprotein, MCH: mean corpuscular haemoglobin, MCHC mean corpuscular haemoglobin concentration; MCV mean corpuscular volume, RBC: red blood cell. (N0 of patients = 97)
I
J
Relative importance
Hemoglobin
Sex
RBC count
Total protein
Albumin
Creatinine
Total bilirubin
Homocysteine
HDL cholesterol
MCH
Alanine aminotraspherase
Alcaline phosphatase
γ-glutamyl carboxilase
Body mass index
Leukocyte count
MCHC
Fibrinogen
Triglyceride
MCV
Platelet count
Hypertension
INR
C-reactive protein
Total protein
Fibrinogen
Alcaline phosphatase
C-reactive protein
γ-glutamyl carboxilase
Platelet count
LDL cholesterol
CeD
Age
Total cholesterol
Albumin
RBC distribution width
Alanine aminostranspherase
INR
Urea
