# Supplementary material for: Diet-Dependent and Diet-Independent Hemorheological Alterations in Celiac Disease: A Case-Control Study
Source: Clin Transl Gastroenterol. 2020 Nov 12;11(11):e00256. doi: 10.14309/ctg.0000000000000256 (PMC7665261; doi:10.14309/ctg.0000000000000256)
Supplement: SUPPLEMENTARY MATERIAL [file ct9-11-e00256-s006.docx]

**Supplemental Digital Content 5. Effects of dietary adherence on hemorheological profile**

**Hemorheological parameters by dietary adherence estimated through coeliac-specific serology**

|  | **Group 1:  seropositive CeD (n=14)** | **Group 2: seronegative CeD (n=36)** | **Group 3: control (n=50)** | **P-values** | | | |
| --- | --- | --- | --- | --- | --- | --- | --- |
|  |  |  |  | **Interaction** | **Group 1 vs 2** | **Group 1 vs 3** | **Group 2 vs 3** |
| Hematocrit (%) | 44.00±4.49 | 43.08±3.29 | 44.44±3.30 | 0.208 |  |  |  |
| Whole blood viscosity (mPa·s) | 4.17±0.57 | 3.99±0.36 | 4.14±0.43 | 0.266 |  |  |  |
| Plasma viscosity (mPa·s) | 1.24±0.16 | 1.24±0.16 | 1.27±0.15 | 0.456 |  |  |  |
| Fibrinogen (g/L) | 2.83 [2.54–3.03]^*^ | 2.98 [2.66–3.78] | 3.16 [2.71–3.59]^*^ | 0.263 |  |  |  |
| Erythrocyte aggregability | | | | |  |  |  |
| AI (%) | 63.63±11.53 | 63.79±9.54 | 64.60±6.33 | 0.879 |  |  |  |
| T_1/2_ (sec) | 2.36±1.28 | 2.29±1.39 | 2.06±0.71 | 0.917 |  |  |  |
| γ (1/sec) | 115.36±75.27 | 103.54±36.82 | 102.25±29.62 | 0.996 |  |  |  |
| Erythrocyte deformability | | | | |  |  |  |
| EI, 30.00 Pa | 0.6250 [0.6163–0.6288] | 0.6255 [0.6220–0.6293] | 0.6290 [0.6260–0.6320] | **0.008** | 1.000 | **0.036** | **0.037** |
| EI, 16.87 Pa | 0.5975 [0.5895–0.6035] | 0.6000 [0.5965–0.6033] | 0.6050 [0.6020–0.6060] | **0.001** | 1.000 | **0.006** | **0.006** |
| EI, 9.49 Pa | 0.5555 [0.5463–0.5610] | 0.5555 [0.5518–0.5600] | 0.5610 [0.5563–0.5640] | **0.002** | 1.000 | **0.043** | **0.006** |
| EI, 5.33 Pa | 0.5010 [0.4930–0.5075] | 0.5015 [0.4958–0.5033] | 0.5060 [0.5000–0.5100] | **0.010** | 1.000 | **0.010** | 0.275 |
| EI, 3.00 Pa | 0.4290 [0.4223–0.4358] | 0.4250 [0.4190–0.4310] | 0.4310 [0.4260–0.4378] | **0.039** | 0.999 | **0.033** | 1.000 |
| EI, 1.69 Pa | 0.3405 [0.3308–0.3513] | 0.3340 [0.3283–0.3465] | 0.3420 [0.3330–0.3470] | 0.363 |  |  |  |
| EI, 0.95 Pa | 0.2390 [0.2200–0.2448] | 0.2260 [0.2158–0.2425] | 0.2280 [0.2280–0.2448] | 0.400 |  |  |  |
| EI, 0.53 Pa | 0.1245 [0.1113–0.1398] | 0.1165 [0.1038–0.1355] | 0.1290 [0.1138–0.1365] | 0.514 |  |  |  |
| EI, 0.30 Pa | 0.0440 [0.0163–0.0550] | 0.0370 [0.0070–0.0495] | 0.0400 [0.0303–0.0505] | 0.658 |  |  |  |

P values<0.05 are highlighted with bold, *indicates unsuccessful measurement for one CeD patient and one control. Parameters are reported in median [Q_1_–Q_3_] or mean ± SD, depending on the distribution. The analysis was done either with one-way ANOVA or with Kruskal-Wallis test (with posthoc Mann-Whitney test). AI: aggregability index; EI: elongation index.

**Hemorheological parameters by dietary adherence estimated through urine-GIP measurement**

|  | **Group 1:  GIP+ CeD (n=6)** | **Group 2: GIP**– **CeD (n=44)** | **Group 3: control (n=50)** | **P-values** | | | | |
| --- | --- | --- | --- | --- | --- | --- | --- | --- |
|  |  |  |  | **Interaction** | **Group 1 vs 2** | **Group 1 vs 3** | | **Group 2 vs 3** |
| Hematocrit (%) | 40.83±5.49 | 43.68±3.25 | 44.44±3.30 | **0.049** | 0.142 | **0.044** | 0.536 | |
| Whole blood viscosity (mPa·s) | 3.83±0.59 | 4.07±0.41 | 4.14±0.43 | 0.180 |  |  |  | |
| Plasma viscosity (mPa·s) | 1.14±0.07 | 1.26±0.16 | 1.27±0.15 | 0.063 |  |  |  | |
| Fibrinogen (g/L) | 2.57 [2.46–2.59]^*^ | 2.95 [2.70–3.78] | 3.16 [2.71–3.59]^*^ | 0.141 |  |  |  | |
| Erythrocyte aggregability | | | | |  |  |  | |
| AI (%) | 57.01±8.24 | 64.67±9.86 | 64.60±6.33 | 0.094 |  |  |  | |
| T_1/2_ (sec) | 3.09±1.45 | 2.21±1.31 | 2.06±0.71 | 0.110 |  |  |  | |
| γ (1/sec) | 102.08±27.95 | 107.50±52.48 | 102.25±29.62 | 0.998 |  |  |  | |
| Erythrocyte deformability | | | | |  |  |  | |
| EI, 30.00 Pa | 0.6250 [0.6165–0.6268] | 0.6255 [0.6218–0.6293] | 0.6290 [0.6260–0.6320] | **0.008** | 1.000 | 0.162 | **0.016** | |
| EI, 16.87 Pa | 0.6010 [0.5858–0.6035] | 0.5995 [0.5948–0.6033] | 0.6050 [0.6020–0.6060] | **0.001** | 1.000 | 0.301 | **0.001** | |
| EI, 9.49 Pa | 0.5550 [0.5395–0.5593] | 0,5555 [0.5500–0.5603] | 0.5610 [0.5563–0.5640] | **0.002** | 1.000 | 0.233 | **0.003** | |
| EI, 5.33 Pa | 0.4965 [0.4838–0.5010] | 0.5020 [0.4960–0.5050] | 0.5060 [0.5000–0.5100] | **0.007** | 0.986 | 0.071 | **0.023** | |
| EI, 3.00 Pa | 0.4170 [0.4060–0.4235] | 0.4260 [0.4198–0.4350] | 0.4310 [0.4260–0.4378] | **0.010** | 0.164 | **0.016** | 0.218 | |
| EI, 1.69 Pa | 0.3210 [0.3123–0.3298] | 0.3370 [0.3300–0.3503] | 0.3420 [0.3330–0.3470] | **0.009** | **0.016** | **0.007** | 1.000 | |
| EI, 0.95 Pa | 0.2075 [0.1903–0.2143] | 0.2380 [0.2178–0.2440] | 0.2280 [0.2280–0.2448] | **0.005** | **0.006** | **0.004** | 1.000 | |
| EI, 0.53 Pa | 0.0910 [0.0660–0.0988] | 0.1260 [0.1100–0.1405] | 0.1290 [0.1138–0.1365] | **0.006** | **0.007** | **0.005** | 1.000 | |
| EI, 0.30 Pa | –0.0260 [–0.0398––0.0025] | 0.0405 [0.0255–0.0530] | 0.0400 [0.0303–0.0505] | **0.003** | **0.002** | **0.003** | 1.000 | |

P values<0.05 are highlighted with bold, *indicates unsuccessful measurement for one CeD patient and one control. Parameters are reported in median [Q_1_–Q_3_] or mean ± SD, depending on the distribution. The analysis was done either with one-way ANOVA (with posthoc Tukey test) or with Kruskal-Wallis test (with posthoc Mann-Whitney test). AI: aggregability index; EI: elongation index; GIP: gluten immunogenic peptide.

**Hemorheological parameters by dietary adherence estimated through dietary interview**

|  | **Group 1:  CeD with poor adherence (n=10)** | **Group 2: CeD with good adherence (n=40)** | **Group 3: control (n=50)** | **P-values** | | | |
| --- | --- | --- | --- | --- | --- | --- | --- |
|  |  |  |  | **Interaction** | **Group 1 vs 2** | **Group 1 vs 3** | **Group 2 vs 3** |
| Hematocrit (%) | 42.10±3.81 | 43.65±3.58 | 44.44±3.30 | 0.133 |  |  |  |
| Whole blood viscosity (mPa·s) | 4.10±0.50 | 4.03±0.42 | 4.14±0.43 | **0.003** | **0.004** | 0.142 | 0.055 |
| Plasma viscosity (mPa·s) | 1.38±0.28 | 1.21±0.09 | 1.27±0.15 | 0.466 |  |  |  |
| Fibrinogen (g/L) | 3.62 [2.90–4.10)^*^ | 2.86 [2.67–3.49] | 3.16 [2.71–3.59]^*^ | 0.213 |  |  |  |
| Erythrocyte aggregability | | | | |  |  |  |
| AI (%) | 71.20±9.22 | 61.89±9.41 | 64.60±6.33 | **0.005** | **0.004** | **0.049** | 0.250 |
| T_1/2_ (sec) | 1.53±0.77 | 2.51±1.39 | 2.06±0.71 | **0.003** | **0.002** | **0.032** | 0.265 |
| γ (1/sec) | 151.75±68.82 | 95.63±37.45 | 102.25±29.62 | **0.002** | **0.001** | **0.010** | 0.405 |
| Erythrocyte deformability | | | | |  |  |  |
| EI, 30.00 Pa | 0.6190 [0.6143–0.6240] | 0.6265 [0.6235–0.6300] | 0.6290 [0.6260–0.6320] | **<0.001** | **0.025** | **<0.001** | 0.143 |
| EI, 16.87 Pa | 0.5915 [0.5875–0.5980] | 0.6010 [0.5970–0.6040] | 0.6050 [0.6020–0.6060] | **<0.001** | 0.157 | **0.001** | **0.012** |
| EI, 9.49 Pa | 0.5505 [0.5438–0.5548] | 0.5570 [0.5518–0.5610] | 0.5610 [0.5563–0.5640] | **0.001** | 0.270 | **0.002** | **0.020** |
| EI, 5.33 Pa | 0.4980 [0.4898–0.5020] | 0.5020 [0.4960–0.5053] | 0.5060 [0.5000–0.5100] | **0.005** | 0.709 | **0.020** | **0.043** |
| EI, 3.00 Pa | 0.4275 [0.4148–0.4308] | 0.4255 [0.4190–0.4350] | 0.4310 [0.4260–0.4378] | 0.054 |  |  |  |
| EI, 1.69 Pa | 0.3405 [0.3233–0.3443] | 0.3350 [0.3290–0.3493] | 0.3420 [0.3330–0.3470] | 0.428 |  |  |  |
| EI, 0.95 Pa | 0.2410 [0.2113–0.2420] | 0.2300 [0.2160–0.2440] | 0.2280 [0.2280–0.2448] | 0.583 |  |  |  |
| EI, 0.53 Pa | 0.1325 [0.1030–0.1398] | 0.1180 [0.1055–0.1373] | 0.1290 [0.1138–0.1365] | 0.574 |  |  |  |
| EI, 0.30 Pa | 0.0495 [0.0135–0.0550] | 0.0350 [0.0108–0.0510] | 0.0400 [0.0303–0.0505] | 0.500 |  |  |  |

P values<0.05 are highlighted with bold, *indicates unsuccessful measurement for one CeD patient and one control. Parameters are reported in median [Q_1_–Q_3_] or mean ± SD, depending on the distribution. The analysis was done either with one-way ANOVA (with posthoc Tukey test) or with Kruskal-Wallis test (with posthoc Mann-Whitney test). AI: aggregability index; EI: elongation index.
