# Supplementary material for: Diet-Dependent and Diet-Independent Hemorheological Alterations in Celiac Disease: A Case-Control Study
Source: Clin Transl Gastroenterol. 2020 Nov 12;11(11):e00256. doi: 10.14309/ctg.0000000000000256 (PMC7665261; doi:10.14309/ctg.0000000000000256)
Supplement: SUPPLEMENTARY MATERIAL [file ct9-11-e00256-s007.pptx]

## Slide 1
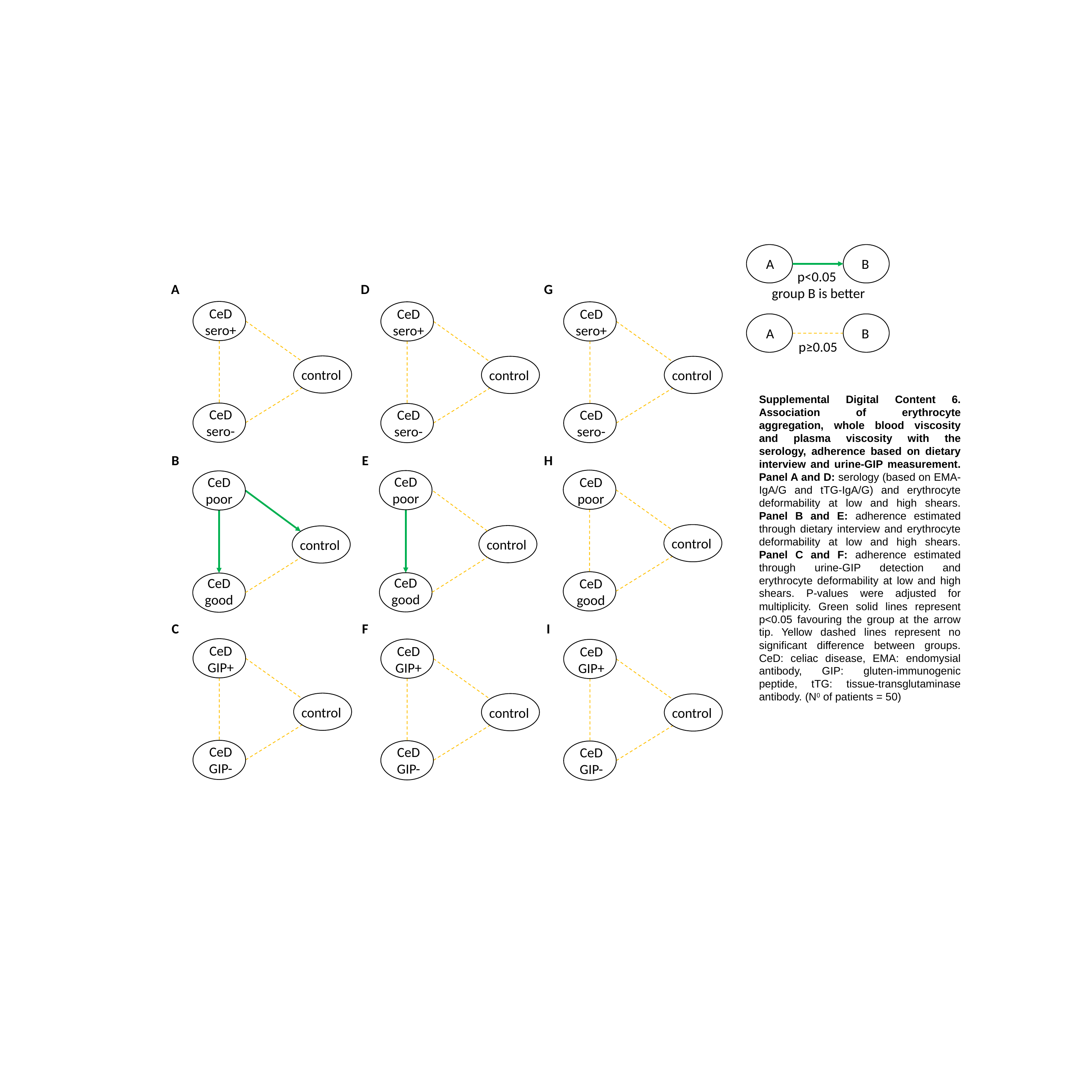

A
B
p<0.05
group B is better
A
D
G
CeD
sero+
CeD
sero+
CeD
sero+
A
B
p≥0.05
control
control
control
Supplemental Digital Content 6. Association of erythrocyte aggregation, whole blood viscosity and plasma viscosity with the serology, adherence based on dietary interview and urine-GIP measurement. Panel A and D: serology (based on EMA-IgA/G and tTG-IgA/G) and erythrocyte deformability at low and high shears. Panel B and E: adherence estimated through dietary interview and erythrocyte deformability at low and high shears. Panel C and F: adherence estimated through urine-GIP detection and erythrocyte deformability at low and high shears. P-values were adjusted for multiplicity. Green solid lines represent p<0.05 favouring the group at the arrow tip. Yellow dashed lines represent no significant difference between groups. CeD: celiac disease, EMA: endomysial antibody, GIP: gluten-immunogenic peptide, tTG: tissue-transglutaminase antibody. (N0 of patients = 50)
CeD
sero-
CeD
sero-
CeD
sero-
B
E
H
CeD
poor
CeD
poor
CeD
poor
control
control
control
CeD
good
CeD
good
CeD
good
C
F
I
CeD
GIP+
CeD
GIP+
CeD
GIP+
control
control
control
CeD
GIP-
CeD
GIP-
CeD
GIP-
